# Perceptions of professional social media interaction with patients and faculty members – a comparative survey among dental students from Malaysia and Finland

**DOI:** 10.1186/s12909-023-04359-1

**Published:** 2023-05-25

**Authors:** Shani Ann Mani, Eswara Uma, Jacob John, Pentti Nieminen

**Affiliations:** 1 Department of Paediatric Dentistry and Orthodontics, Faculty of Dentistry, Universiti Malaya, Kuala Lumpur, 50603 Malaysia; 2 Department of Pediatric Dentistry, Faculty of Dentistry, Manipal University College Malaysia, Melaka, 75150 Malaysia; 3 Department of Restorative Dentistry, Faculty of Dentistry, Universiti Malaya, Kuala Lumpur, 50603 Malaysia; 4 Medical Informatics and Data Analysis Research Group, University of Oulu, Oulu, 90014 Finland

**Keywords:** E-professionalism, Social media usage, Dental undergraduate students

## Abstract

**Background:**

Professional and personal boundaries are blurred with the wide application of social media (SM) in the health professions line of work. Little is known about practice of extending friend requests to patients and faculty members among dental students, which encompass a part of E-professionalism. The aim of this study is to assess the factors associated with the perceptions and practices of interactions with patients and faculty on SM among dental students from Malaysia and Finland.

**Methods:**

Dental students from 4 institutions in Malaysia and Finland completed self-administered questionnaires on the practices and perceptions of SM use. The main variables assessed were the perceptions and practices of student-patient and student-faculty communication on SM, between the two countries. Students’ country, age, gender, time spent on SM and perceived importance of communicating dental related aspects over SM were analysed as potential explanatory variables. Crosstabulation was used to estimate the distributions of the response variables by the background characteristics. Multivariate analyses were performed using a dichotomous logistic regression model to investigate relevant associations between the responses and the explanatory variables independent from other factors.

**Results:**

A total of 643 students completed the survey in March-April 2021. More Malaysian students agreed with “guiding patients online is a new responsibility for dentists in the digital age” compared to Finnish students (86.4% vs. 73.4%). Similarly, significantly more Malaysian students befriended patients (14.1% vs. 1%) and invited faculty to be friends on SM (73.6% vs. 11.8%). Expectedly, clinical year students befriended patients more than pre-clinical (13.8% vs. 6.8%). Significantly more students who felt ‘communication of dental related issues over SM’ were likely to extend friend requests to faculty rather than accept patient friend requests.

**Conclusions:**

Social media regulations and socio-cultural practices contribute to dental students’ attitudes and behaviour when befriending patients and faculty members on social media. Future dental curriculum should incorporate guidelines for professional communication on social media based on local and cultural needs. Students’ should be encouraged to interact with their patients using professional identities on social media.

## Background

Social media (SM) usage among health professions’ students is an established norm, with dentistry being no exception [1–3]. On SM, students’ construct public or semipublic profiles, either personal or related to their field-of-study, or a combination of both, enabling them to create, circulate, share, and exchange information with their connections [4]. Generally, accepting friend requests over SM is less judicious and more spontaneous [5]. In a rapidly changing world, where SM is playing an important role, there is a blurring of boundaries between professional and personal lives when health professions’students connect with patients and faculty members on SM [6, 7]. Concurrently, this poses professional and ethical challenges to doctor-patient and student-faculty relationships at large with differences of opinion regarding professional boundaries [8].

Today’s patients seek support for their healthcare needs through various online sources and are likely to connect with their healthcare providers through SM [9]. While this is beneficial for patients, such communication outside working hours is demanding in terms of time and physical energy, thus disrupting study-life balance of health professions’students [6, 10]. Casual online postings by students in health care related to patients, peers or faculty may be shared with a wider audience thus violating the privacy rights of the individual. [6, 10]. Additionally, connecting with patients on SM is likely to expose the details of personal lives of students and their online connections, risking reputations, safety and privacy [6, 10]. Students should be encouraged to have separate personal and professional content online, if SM interactions with patients is permitted by the institution [11].

In most countries, dental regulatory bodies have outlined clear guidelines for SM use among professionals and dental students [12, 13]. However, many of the guidelines do not specifically address friend requests from patients and adding faculty members as friends. Students’ preferences on whether to connect with patients and faculty vary widely [2, 3, 14]. Generally, students were anxious about the possibility of faculty reading personal posts in SM [7]. Much of the practice is based on perceived notions of the students and the society-driven practices in the region [2, 3, 14].

In a digital era, students should be prepared to develop a professional identity online to share professionally related content and to communicate with patients and faculty via SM. This will shape and define their future professional practices and adapt to the updated SM use regulations laid out by the regulatory bodies. E-professionalism is a more recent concept and although implied, may not be explicitly taught in the curriculum of all dental schools. Currently, there is limited knowledge on factors affecting dental students’ perceptions and practices of interactions with patients and faculty on SM in countries with wide socio-cultural diversity such as Malaysia and Finland. Hence, our null hypothesis was that dental students’ perceptions and practices of interactions with patients and faculty on SM will not differ based on location, age, sex, year of study, hours spent on social media and importance given to communicating issues related to dental training via SM. We reported the findings regarding SM usage and e-professionalism among dental undergraduate students in Malaysia and Finland [1, 15]. Although the same five SM platforms (WhatsApp, YouTube, Instagram, Facebook, and Snapchat) were used in both countries, e-professionalism practices differed remarkably between the two countries suggesting the influence of socio-cultural diversity. The aim of this study was to assess the factors associated with the perceptions and practices of interactions with patients and faculty on social media among dental students from Malaysia and Finland.

## Methods

### Study design and data collection

All undergraduates enrolled in the 5-year dentistry course from two dental schools in Malaysia (Manipal University College Malaysia and Universiti Malaya) and Finland (University of Helsinki and University of Oulu) each were invited to participate in this study during the academic year 2020–2021. The study population size (number of dental undergraduates of the academic year 2020–2021) was about 3250 in Malaysia and 1000 in Finland. The anonymous Google forms questionnaire was circulated via email and WhatsApp between March 23rd, 2021 and April 11th, 2021 in both countries. All students were required to read the Participant information sheet (PIS) and give consent for data collection and processing. The participation was blocked if the student refused consent. To ensure no data was missing, the online survey was designed such that all items had to be answered prior to submission of the form. This study was part of a larger survey involving the same respondents and hence the sample size calculation was based on our previous study of dental students’ use of social media [1]

Ethical approval to conduct this study was obtained only in Malaysia; Medical Ethics Committee, Faculty of Dentistry, Universiti Malaya [DF CD2105/0015 (L)] and Research Ethics committee, Faculty of dentistry, Manipal University College Malaysia [ MMMC/FOD/AR/E C-2021(F-01)]. In Finland, the Ministry of Education and Culture has exempted surveys conducted with anonymous questionnaires from obtaining approval by an ethics committee. The study followed the Finnish National Board on Research Integrity TENK’s guidelines on ethical principles.

The instrument used was a questionnaire modified from a previous study among medical students in the USA [16]. Questionnaire items were presented in three sections; Part A addressed the demographic characteristics of the participants. Part B focused on their use and time spent on various SM platforms. Part C concentrated on practice and perceptions regarding e-professionalism. Most questions required responses on a 4-point or 5-point Likert scale. Prior to the start of our study, the English questionnaire was pre-tested on a sample of five students in different years to check for semantic comprehension in both institutions in Malaysia, resulting in some minor modifications. In Finland, the translated Finnish version was also pre-tested on five dental students, following which minor changes were made to improve the language and to clarify the purpose of the questions. In this paper, we analysed the factors associated with three outcome variables that addressed the perceptions and practices about guiding patients online, accepting invitations from patients to be “friends”, and inviting faculty members to be “friends” between the two countries. The attitude to SM interaction for guiding patients was assessed by the question ‘‘Do you agree or disagree with the statement that guiding patients online is a new responsibility for dentists in the digital age?”. SM invitation activity was assessed by two questions: “I have accepted invitation(s) from patients to be “friends"”. and “I have invited faculty members (my institution/ other institutions) to be “friends"”. Response alternatives were: no, yes.

### Statistical analysis

**Fig. 1:**
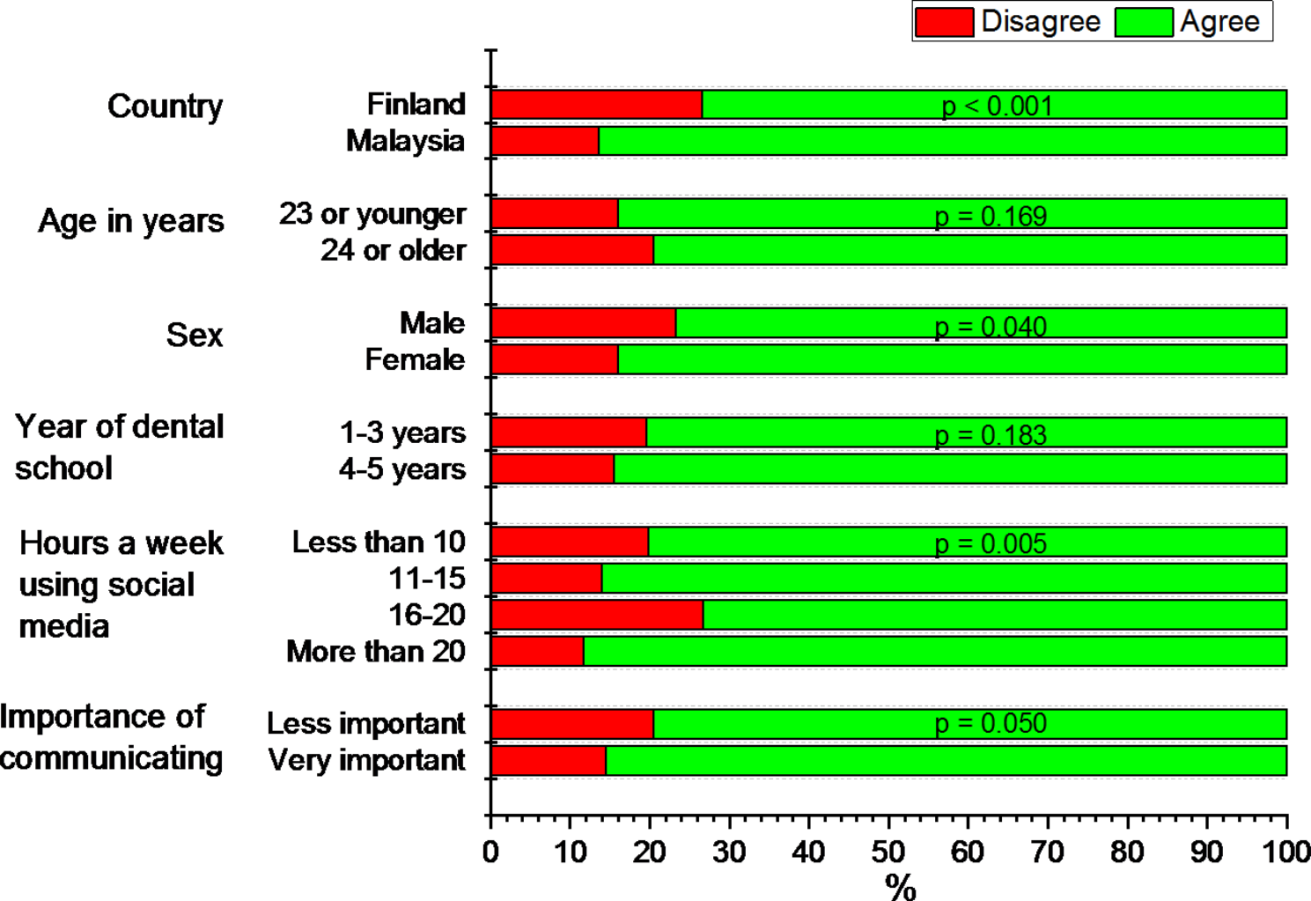
Percentage distributions of responses to the question “Do you agree or disagree with the statement that guiding patients online is a new responsibility for dentists in the digital age?“ by chosen factors. Data include dental undergraduate students from Malaysia (n = 440) and Finland (n = 203). Statistical significances between the countries were evaluated by chi-square test. Variable “Importance of communicating” measures responses to a question “How important is communicating about issues relating to dental training in encouraging you to use social media?”

The data were analysed using IBM SPSS for Windows (version 26) (IBM Corp. Armonk, NY, USA) and Origin 2020 graphing software (OriginLab, Northampton, MA, USA). The frequency and percentage distributions were used to present the background characteristics (country, age, sex, year of dental school, hours a week using SM, and attitude about importance of communicating issues relating to dental training) of the participants. Based on age, participants were grouped into two categories; “23 or younger” and “24 or older” to facilitate and clarify the presentation of the relationship between response variables and age of students. The cut-off value 24 years was chosen as the median age of the whole student sample. Crosstabulation was used to estimate the distributions of the outcome variables by the background characteristics. In the cross-tabulation, statistical significance of the association between the responses and explanatory variables were evaluated with the chi-squared test. The distribution of attitudes to guiding patients online are also illustrated in Fig. 1. Multivariable analyses were performed using a dichotomous logistic regression model to investigate relevant associations between the outcome variables and the explanatory variables independent from other factors. The logistic models were reported using adjusted odds ratios (OR) and their 95% confidence intervals (CI).

## Results

### Participants

A total of 591 and 487 students were invited from Malaysia and Finland respectively to participate in the survey. The study participants consisted of 643 undergraduate dental students from Malaysia (n = 440) and Finland (n = 203) representing a response rate of 74.4% and 41.7% respectively. Their basic characteristics are shown in Table 1. The students were mostly female and younger than 24 years. Just over half of the students (54.9%) were in the early stages of their studies (1–3 years). Most of the students spent more than 11 hours per week using SM applications.

**Table 1:** The frequency and percentage distributions of basic characteristics among 643 dental students from Finland and Malaysia

Variables	n (%)
Country	
Finland	203 (31.6)
Malaysia	440 (68.4)
Age	
23 or younger	393 (61.1)
24 or older	250 (38.9)
Sex	
Male	150 (23.3)
Female	493 (76.7)
Year of dental school	
1–3 years	353 (54.9)
4–5 years	290 (45.1)
Hours a week using SM	
Less than 10 h	217 (33.7)
11–15 h	143 (22.2)
16–20 h	120 (18.7)
More than 20 h	163 (25.3)
Importance of communicating issues relating to dental training	
Less important	347 (54.0)
Very important	296 (46.0)

### Perceptions about guiding patients online

A total of 529 (82.3%) students agreed with the statement that guiding patients online is a new responsibility for dentists in the digital age. Figure 1 shows that a higher percentage of Malaysian students agreed with this statement compared to Finnish students (86.4% vs. 73.4%), p-value of chi-square test < 0.001). Responses to the statement were also statistically significantly associated with the gender and time spent on using SM (entertainment, communication, learning and searching information). In addition, probability to agree with the statement was higher among students who felt it important to communicate about issues relating to dental training (p = 0.0497). Multivariable logistic regression analysis revealed that the country of the student’s dental school and time spent on SM were the most important (only independent) factors to explain the perception / agreement on the new responsibility for dentists to guide patients online (Table 2).

**Table 2:** Factors, their adjusted odds ratios and 95% confidence intervals for predicting the students’ agreeing attitude to responsibility to guide patients online using multivariable logistic regression analysis. Data included 643 undergraduate dental students

	Responsibility to guide patients		
	Odds ratio	95% CI	p-value
Country			0.003
Finland (ref)	1		
Malaysia	2.17	1.31–3.59	
Age			0.664
23 or younger	1.13	0.65–1.96	
24 or older (ref)	1		
Sex			0.061
Male (ref)	1		
Female	1.57	0.99–2.50	
Year of dental school			0.333
1–3 years (ref)	1		
4–5 years	1.28	0.77–2.12	
Hours a week using SM			0.006
Less than 10 h (ref)	1		
11–15 h	1.81	0.99–3.29	
16–20 h	0.75	0.44–1.29	
More than 20 h	1.91	1.06–3.46	
Importance of communicating issues relating to dental training			0.205
Less important (ref)	1		
Very important	1.32	0.86–2.02	

### Invitations from patients

Table 3 presents the distribution of accepting online invitations from patients to be “friends” by the analyzed factors. A total of 64 students (10.0%) had accepted invitations. The proportion was statistically significantly higher among Malaysian students. Dental students with more years of study had accepted invitations more often than students with only 1–3 years of study. These associations remained significant also when controlled for other factors under study in the logistic regression analysis (Table 4). Compared to Finnish students, Malaysian students were more likely to accept invitations (adjusted OR 12.03, 95% CI = 2.80 -51.72). In bivariable analysis, students who emphasized the importance of communicating issues related to dental education had responded more often to invitations. However, this effect did not remain as an independent factor by the logistic regression analysis (OR 1.62, 95% CI = 0.94–2.79). Results also indicated a trend toward a statistically significant finding that female students accepted invitations from patients less often.

**Table 3:** Distributions of response variable “I have accepted invitation(s) from patients to be “friends”" by the characteristics of the dental undergraduate students from Finland and Malaysia

Variables	No n (%)	Yes n (%)	Total	P-value of chi-square test
Country				< 0.001
Finland	201 (99.0)	2 (1.0)	203 (100)	
Malaysia	378 (85.9)	62 (14.1)	440 (100)	
Age				0.112
23 or younger	348 (88.5)	45 (11.5)	393 (100)	
24 or older	231 (92.4)	19 (7.6)	250 (100)	
Sex				0.059
Male	129 (86.0)	21 (14.0)	150 (100)	
Female	450 (91.3)	43 (8.7)	493 (100)	
Year in dental school				0.003
1–3 years	329 (93.2)	24 (6.8)	353 (100)	
4–5 years	250 (86.2)	40 (13.8)	290 (100)	
Hours a week using SM				0.058
Less than 10 h	190 (87.6)	27 (12.4)	217 (100)	
11–15 h	134 (93.7)	9 (6.3)	143 (100)	
16–20 h	113 (94.2)	7 (5.8)	120 (100)	
More than 20 h	142 (87.1)	21 (12.9)	163 (100)	
Importance of communicating issues relating to dental training				0.024
Less important	321 (92.5)	28 (7.5)	347 (100)	
Very important	258 (87.2)	38 (12.8)	296 (100)	
Total	579 (90.0)	64 (10.0)	643(100)	

**Table 4:** Factors, their adjusted odds ratios and 95% confidence intervals for predicting “I have accepted invitation(s) from patients to be “friends”" and to “I have invited faculty members (my institution/ other institutions) to be “friends”" using multivariable logistic regression analysis. Data included 643 undergraduate dental students

	I have accepted invitation(s) from patients to be “Friends”					I have invited faculty members (my institution/ other institutions) to be “Friends”					
	Odds ratio	95% CI	P-value			Odds ratio			95% CI		p-value
Country			< 0.001								< 0.001
Finland (ref)	1					1					
Malaysia	12.03	2.80 -51.72				20.29			11.76–35.02		
Age			0.359								0.884
23 or younger	1.38	0.69–2.76				1.04			0.61–1.78		
24 or older (ref)	1					1					
Sex			0.074								0.310
Male (ref)	1					1					
Female	0.58	0.32–1.04				0.79			0.50–1.24		
Year of dental school			0.008								0.355
1–3 years (ref)	1					1					
4–5 years	2.36	1.25–4.46				1.25			0.78–2.01		
Hours a week using SM			0.159								0.271
Less than 10 h (ref)	1						1				
11–15 h	0.59	0.25–1.26				0.78			0.46–1.33		
16–20 h	0.49	0.20–1.20				1.27			0.72–2.26		
More than 20 h	1.09	0.58–2.06				0.75			0.46–1.23		
Importance of communicating issues relating to dental training			0.083								0.011
Less important (ref)	1					1					
Very important	1.62	0.94–2.79				1.65			1.12–2.44		

### Inviting faculty members

We also analyzed the statement “I have invited faculty members (my institution/ other institutions) to be “friends”" (No, Yes) as a response variable. More than half (54.1%) of the participants had invited faculty members to be “friends” (Table 5). Students from Malaysia, younger age, and those who emphasized the importance of communicating issues related to dental training reportedly had more frequently invited faculty members to be friends on SM. In addition, the number of hours a week spent on SM was significantly associated with this response variable (p = 0.033). To investigate the independent effect of these variables on inviting faculty members, we performed multivariable binary logistic regression analysis. Only country and importance of communicating issues relating to dental training remained as statistically significant factors for sending friend invitations to faculty. Malaysian dental students had higher probability to invite faculty members (OR 20.29, 95% CI = 11.76–35.02). Those who believed communicating issues relating to dental training on SM as very important were more likely to extend a friend invitation to faculty on SM (OR 1.65, 95% CI = 1.12–2.44). Age, sex, year in dental school and hours a week using SM had no significant independent effect on the response variable.

**Table 5:** Distributions of response variable “I have invited faculty members (my institution/ other institutions) to be “friends”" by the characteristics of dental undergraduate students from Finland and Malaysia

Variables	No n (%)	Yes n (%)	Total	P-value of chi-square test
Country				< 0.001
Finland	179 (88.2)	24 (11.8)	203 (100)	
Malaysia	116 (26.4)	324 (73.6)	440 (100)	
Age				< 0.001
23 or younger	148 (37.7)	245 (62.3)	393 (100)	
24 or older	147 (58.8)	103 (41.2)	250 (100)	
Sex				0.475
Male	65 (43.3)	85 (56.7)	150 (100)	
Female	230 (46.7)	263 (53.3)	493 (100)	
Year in dental school				0.017
1–3 years	177 (50.1)	176 (49.9)	353 (100)	
4–5 years	118 (40.7)	172 (59.3)	290 (100)	
Hours a week using SM				0.033
Less than 10 h	86 (39.6)	131 (60.4)	217 (100)	
11–15 h	79 (55.2)	64 (44.8)	143 (100)	
16–20 h	53 (44.2)	67 (55.8)	120 (100)	
More than 20 h	77 (47.2)	86 (52.8)	163 (100)	
Importance of communicating issues relating to dental training				0.001
Less important	181 (52.2)	166 (47.8)	347 (100)	
Very important	114 (38.5)	182 (61.5)	296 (100)	
Total	295 (45.9)	348 (54.1)	643(100)	

## Discussion

Today’s health professions’ students face the challenge of balancing their online professional and private persona. Being a novice in the professional field, often times students continue their previous practices and may not be aware of the consequences of online activity and the professional way to conduct themselves online [14, 17]. Traditionally, the student-patient and student-faculty professional or informal interactions were limited to face-to-face interactions, either inside or outside of the clinic or classroom. With SM use, student- patient online interactions and can occur at all hours and can be problematic, as they are not yet professionals, while simultaneously being expected to abide by ethical and professional rules [3]. Guidelines are in place in most countries and institutions, but do not address the potential repercussions of student- patient interactions. In the UK, the General Dental Council (GDC) guidelines for professionals’ state that careful consideration is needed before adding patients as friends. Conversely, dental students expressed the need for autonomy with regards to online activity, not having to adhere to guidelines or institutional authority as a student [3, 18].

Perceptions towards student-patient interactions vary between countries with about 40-50% of dental students being willing to befriend patients on SM in Saudi Arabia and Greece [2, 3, 17] while in the UK, such interactions were considered appropriate only by a few [14]. Although only 10% of students in our study accepted ‘friend’ requests from patients, the socio-cultural differences between the two countries may explain the statistically significant difference, where 14% of Malaysian students had accepted ‘friend’ requests from patients, whereas only 1% had done so in Finland. Moreover, the survey was conducted during the COVID pandemic when both countries had severe restrictions, and SM played an important role in both societies. Exceptional circumstances during the pandemic may therefore not explain the differences. However, one reason for the difference might be the strict legislation on the European Union area (including Finland) concerning the individuals’ data protection. Online interaction (“friending”) with a patient is generally not acceptable nor endorsed.

A higher percentage of dental students were likely to be friends with patients when compared to medical students [3, 7]. These differences are attributed to dental profession using SM as a marketing tool (pre and post photographs of patients) and being driven by esthetic outcomes [3, 19]. About two-third of dental students in Saudi Arabia believed that routine posting on SM increases their market value [2] Dental students exposed to these practices early on are likely to perceive this to be an acceptable norm.

A higher percentage of fifth-year dental students (48.3%) compared to fourth-year students (20.6%) had received Facebook friend request from patients [17]. A similar finding was noted in this survey where significantly more clinical year 4 and 5 students had accepted friend requests from patients. This is expected as pre-clinical dental students were yet to be assigned patients, while clinical year dental students work closely with their patients on repeated follow up visits for more complex treatment needs, thereby developing a closer bond with increased likelihood to be connected on SM [3]. However, this difference was not significant when younger age groups (< 23 years) were compared to the older age groups in our study perhaps due to minor age differences between the groups. This suggests that familiarity with patients may be the primary reason for students accepting friend requests. In studies where students were compared to senior faculty members or interns, the pattern suggested that younger age groups are more open to being friends with patients [2, 5].

The more time students spent on SM, the more likely they were to add patients as friends and agree that ‘guiding patients’ online is a new responsibility for dentists in this study. It is only natural for this generation of students to communicate all aspects of patient care online since they spend much time online. The implications of such patterns of communication are varied and need to be studied in future. For example, advice for one patient may be shared inadvertently with others and risk being misinterpreted.

Our findings indicate a trend toward a statistically significant effect of sex on perceptions about guiding patients online and accepting invitations from patients, but not regarding inviting faculty members. Female students seemed to agree more often that guiding patients online is a new responsibility for dentists in the digital age. Studies indicate gender-based differences where female healthcare workers may provide preventive care more often, use more patient-centered communication, and provide more psychosocial counselling to their patients than do their male peers [20, 21] However, female students accepted invitations from patients to be “friends” less often, perhaps because they may be more cautious and reluctant to share their personal space with acquaintances.

The intended reasons for students who extended friend requests to faculty members in this study was not investigated. Significantly twenty times more Malaysian dental students extended friend requests via SM to faculty members compared to Finnish dental students. This could be attributed to varying practices between the two regions and different perceptions of ‘friending’ faculty members via SM, and in the absence of guidelines, individuals tend to follow their personal instincts regarding the matter [5, 22]. As anticipated, increased hours on SM did not increase the chances of extending friend invitations to faculty in this study. About 2/3rd of the first-year dental students in the UK believed that connections with academic staff should remain strictly professional during the course of study and friend requests should not be extended to academics [14]. Some students felt forced to accept friend requests from faculty by virtue of being polite [14]. On the other hand, more than 50% of students in Pakistan considered it appropriate to extend a friend request to a faculty member [5].

Generally medical and pharmacy faculty members did not find it appropriate to be friends with current students but are open to connecting with former students [5, 23]. Only 15% of dental faculty in the US used SM to communicate with students [24]. The main concerns among both students and faculty regarding connecting with the other on SM was the blurring of professional boundaries [25]. However appropriate privacy settings and creating professional identities on SM can overcome the disadvantage of blurring professional boundaries to take advantage of the benefits of SM connections between students and faculty [5]. Additionally, well-delineated and unambiguous policies for both students and faculty will guide current practices [26].

Most educational institutions have online platforms that allow two-way communications between students and faculty outside class hours, allowing sharing of posts, links, discussion, audio-visual resources etc. There are many benefits for faculty-student connections on SM such as mentoring, giving additional access to students to address queries prior or after class over and above the traditional instruction [5, 24]. This is especially valuable for students who have inhibitions during class hours, but feel comfortable to engage on SM. A closed Facebook discussion group for pre-clinical medical education enabled rapport with faculty, content learning, and improved emotional well-being among students [27]. In this study, those who perceived the importance of communicating issues with regards to dental training via SM were 1.65 times more likely to extend friend requests to faculty members. Only a third of dental students surveyed in the US believed that SM should be incorporated in their courses [25] and similarly one third of dental faculty in the US perceived no use of SM in teaching in 2014 [24]. More recently however, use of SM has become a part and parcel of a dental student life [14], but being connected with academics over SM remain the top 5 concerns of SM use among students and faculty in Saudi Arabia [28, 29].

Therefore, training students about e-professionalism as part of the undergraduate dental curriculum is important to establish professional practices [30]. The “brown envelope” intervention was well received by students in the UK regarding privacy settings in SM [18]. Students felt regular reminders were required during the course of the curriculum regarding aspects of digital professionalism [18]. Similar techniques can be used to train students on the policy regarding extension of friend requests to patients and faculty as established at the respective institution.

This study is not without limitations. Being a survey, there were limitations in sample selection, where those with biases may have selected themselves into the sample. Secondly, not all institutions were taken into consideration in Malaysia. Lastly, the questionnaire used in this survey was piloted to check for semantic comprehension and adaptability in the current population, but not rigorously validated.

## Conclusions

The practice of ‘friending’ patients and faculty members varied significantly between Malaysia and Finland. Students in the clinical years were more likely to develop friendships with patients over SM. Students were more likely to extend friend requests to faculty members than accept friend requests from patients. Students who believed in communicating issues regarding dentistry were more likely to extend friend requests to faculty over SM. Implications include preparing future students on E-professionalism as per the social milieu and SM guidelines prepared for the region.

## Data Availability

The datasets used and/or analysed during the current study are available from the corresponding author on reasonable request.

## Abbreviations

SM: Social Media
SPSS: Statistical Package for the Social Science
